# From Nutritional Adequacy to Hygiene Quality: A Detailed Assessment of Commercial Raw Pet-Food for Dogs and Cats

**DOI:** 10.3390/ani12182395

**Published:** 2022-09-13

**Authors:** Carla Giuditta Vecchiato, Karin Schwaiger, Giacomo Biagi, Britta Dobenecker

**Affiliations:** 1 Department of Veterinary Medical Sciences, University of Bologna, via Tolara di Sopra 50, 40064 Ozzano Emilia, Italy; 2 Unit of Food Hygiene and Technology, Institute of Food Safety, Food Technology and Veterinary Public Health, University of Veterinary Medicine Vienna, Veterinärplatz 1, 1210 Wien, Austria; 3 Chair of Animal Nutrition and Dietetics, Department of Animal Sciences, Ludwig-Maximilians-Universität Munich, Schönleutnerstr. 8, D85764 Oberschleissheim, Germany

**Keywords:** raw pet food, unconventional pet food, pet nutrition, nutritional deficiencies, high-fat diets, pet food hygiene

## Abstract

**Simple Summary:**

As alternative diets to processed pet food, raw meat-based diets (RMBDs) have received increasing attention in recent years. Commercial RMBDs consist mainly of raw meats, animal by-products, such as offal, and bones. Most RMBDs do not contain additives; therefore, they potentially pose a risk of nutritional imbalances to adults and, to a greater extent, growing animals. RMBDs are frozen ready-to-use products that are fed to pets after being thawed. Bacterial contamination of RMBDs is an area of concern, including the risk of spreading zoonotic diseases to humans. In this study, a detailed assessment of commercial RMBDs was performed in relation to nutritional adequacy and hygiene quality. The results showed that the tested products often provided deficient amounts of essential nutrients, were nutritionally unbalanced, and very rich in fat. They were also characterized by high microbial contamination, showing low hygiene quality. This study provides information that indicates the need for strict monitoring of the nutritional and microbiological quality of commercial RMBDs.

**Abstract:**

Raw meat-based diets (RMBDs) are widely used as unconventional diets for dogs and cats at different life stages, despite concerns regarding nutritional adequacy and microbial contamination. The aim of this study was to evaluate both the nutritional and hygiene quality profile of RMBDs purchased in Germany. For this purpose, crude nutrients were assessed in 44 RMBDs and compared to declared values. In addition, selected minerals were determined in 31 RMBDs labelled as complete and compared to the minimum requirement (MR) for intended species and life stages. Aerobic colony count (ACC) and *Enterobacteriaceae* were used to assess the hygiene quality of 37 commercial RMBDs, while the presence of *Salmonella* spp. was examined in 10 products. Fat and protein content exceeded tolerated deviation from declared values in 33% and 45% of RMBDs, respectively. Each RMBD showed at least one concern regarding nutrient content. The RMBDs had high fat contents (mean 69, range 33–95 g/Mcal) that were negatively correlated with protein (r = −0.74, *p* < 0.0001). Considerable contaminations by ACC and *Enterobacteriaceae* were found (2.61 × 10^8^ ± 3.63 × 10^8^ and 3.61 × 10^6^ ± 8.39 x10^6^ CFU/g, respectively). A higher count of *Enterobacteriaceae* was detected in a frozen RMBDs made of poultry or carcasses from different animals, compared to the thawed counterpart (*p* = 0.003), as well as compared to other sources, such as beef, horse, and lamb, regardless of the storage condition. *Salmonella* spp. were found in 2/10 RMBDs. This study confirmed that feeding commercial RMBDs might pose a risk to pet health.

## 1. Introduction

In dogs and cats, a state of well-being and healthcare depends to a considerable degree on appropriate nutrition [1,2]. The primary purpose of a diet is to provide the amount of energy and nutrients to meet the animal’s requirements, and complete diets, by definition, do not require further supplementations for this purpose [2,3]. Complete commercial pet food is by far the most commonly used feeding method in dogs and cats [4], also including more recent types of commercial unconventional diets such as raw meat-based diets (RMBDs).

Nowadays, RMBDs are increasing in popularity among pet owners who want to avoid processed pet food, preservatives and additives [5,6]. In addition to the perception of RMBDs as a healthier and more natural feeding option than conventional pet food, pet owners might wrongly assume that the quality of commercial diets is consistently good [4]. In a recent study, in fact, none of the products that were evaluated provided all selected macronutrients in adequate concentrations [7], potentially causing malnutrition. Plausible explanations lie in inadequate formulations as well as the high variability of raw materials [5,8]. The latter can result in a very high fat content of RMBDs, with wide deviations between declared and real values [9].

RMBDs are thermally unprocessed, therefore pathogenic bacteria contaminating those products are not rendered inert before being fed to pets. The risk of transmission of pathogens to animals through RMBD is well documented in the literature [8,10,11,12]. For instance, *Salmonella* contamination has been confirmed with serious levels of prevalence between 20 and 70% [11,12,13]. Moreover, the overall hygiene quality is a matter of concern in RMBDs, with more than 50% of evaluated products found to exceed the regulatory threshold for *Enterobacteriaceae* in raw pet food [10,14,15]. Feeding RMBDs may therefore pose a risk to both animal and public health, especially for immunocompromised people or those with severe illnesses. For this reason, they have been banned in certain training programs for therapy dogs [16,17]. The purpose of this study was to perform a comprehensive assessment of both the nutritional and hygiene quality profile of RMBDs purchased in Germany from different major online retailers. Products for different species and life stages were purchased as well as products containing different animal meat and by-product (ABPs) sources.

## 2. Materials and Methods

### 2.1. RMBD Sampling and Processing

A nutrient and microbiological evaluation of frozen commercial RMBDs was performed. Forty-four samples of frozen RMBDs for either dogs or cats were purchased in 2017. All samples were selected from 3 different German internet top-sellers for such products. To minimize the influence of different shipping conditions, all samples were purchased at the same time and similar shipping-time options (delivery guaranteed within 48–72 h after being shipped) were chosen. Immediately after receiving the delivery, the RMBDs were sampled and prepared for analyses of both microbiological quality and nutrient content. All samples were processed, on average, 509 ± 182 days before their expiry dates, which was calculated by subtracting the expiry date of each sample from the day of analyses). An uninterrupted cold chain (≤−20 °C) was guaranteed until analyses.

RMBD product labels were checked to verify compliance with the following mandatory labelling information on pet food labels (Commission Regulation (EC) No 767/2009) [18]): (1) animal species and life stages in case of products labelled as complete (growth, reproduction, maintenance), (2) type of RBMD (complete/complementary pet food), (3) the presence/absence of hygiene and handling advice, and (4) composition (ingredients list).

#### 2.1.1. Nutrient Content Analysis

RMBDs (*n* = 44) subjected to chemical analyses were freeze-dried, ground, homogenised and sampled. Nutritional composition was determined by Weende analyses, performed in duplicate (VDLUFA, 2012 [19]). The analytical constituents (crude fat, crude protein, crude ash, crude fibre) and moisture of each RMBD were compared to the declared composition according to the tolerances permitted by the Commission Regulation (EC) No 767/2009 (Annex IV, part A) [18]. Data are given as median and range.

In addition, 31 RMBD products labelled as complete were analysed for calcium, phosphorus, zinc and copper. For mineral analyses, samples underwent wet digestion with 65% HNO3 in a microwave digestion unit (Ethos 1600, MLS GmbH Leutkirch, Germany). Calcium was determined by flame emission spectrometry (EFOX 5053, Eppendorf AG, Hamburg, Germany) and phosphorus photometrically (GENESYS 10 UV, Thermo Spectronic, Rochester, NY, USA) using the modified vanadate molybdate method modified according to Gericke and Kurmies (1952) [20]. Zinc and copper were measured by atomic absorption spectrometry.

Gross energy (GE) was experimentally determined by adiabatic bomb calorimetry (IKA^®^ Kalorimetersystem C2000), and metabolizable energy (ME) was estimated using analysed GE according to European standard EN 16967:2017 (E) [21].

Protein, fat, calcium, phosphorus, zinc and copper content (expressed as g nutrient/Mcal) was then compared with the minimum recommended nutrient level provided by FEDIAF (2021) [3], according to the age phase the products were intended for (adult, growth or both). For adult animals, a MER of 95 kcal/kg^0.75^ and 75 kcal/kg^0.67^ was considered for dogs and cats, respectively. Early growth (<14 weeks) and late growth (>14 weeks) were considered for complete RBMDs intended for growth, while late growth and adult requirements were considered for RMBDs labelled as adequate to fulfil requirements for all life stages of the species. In addition to cat adult MER, kitten growth stage was also taken into account for RMBDs labelled as adequate to fulfil requirements for maintenance and growth.

#### 2.1.2. Microbiological Analyses

A total of 37 RMBDs were thawed at room temperature before being assessed for hygiene quality.

The following microbiological investigations were performed:

- The quantification of aerobic bacteria was determined by the surface plating technique following the Official Method of Analysis (DIN EN ISO 4833-2:2013).
- The quantification of *Enterobacteriaceae* was determined according to ISO (21528-2:2017).
- In addition, 10/37 products were randomly selected among different complete and complement single-protein ABP RMBDs to determine the presence of *Salmonella* species according to the horizontal method for the detection of *Salmonella* spp. in food and animal feeding stuffs (ISO 6579:2017), in conjunction with an automated enzyme-linked immunosorbent assay method in 2 pooled samples from 5 different RMBDs each (VIDAS^®^ LIS, BioMerieux, Durham, NC, USA). In case of a positive result in the pooled samples, the investigation was repeated for every single sample.

The results of the microbiological analyses were then evaluated. EC No 142/2011—Annex XIII—Chapter II [22] regulates raw pet food regarding maximum values for *Enterobacteriaceae* and *Salmonella* spp. According to the composition and handling habits of those raw products, the following standards were considered related to the overall bacterial contaminations: (1) EC No 2073/2005 on microbiological criteria for foodstuffs, process hygiene criteria for meat and products thereof, as regards aerobic colony count; (2) microbiological critical issues and warning values for the evaluation of food (recommendations by the German Society for Hygiene and Microbiology—DGHM e.V.) regarding *Enterobacteriaceae.*

#### 2.1.3. Statistics Analysis

Results were analysed by descriptive statistics (Excel, Microsoft) and data are given as mean ± standard deviation unless otherwise indicated. Pearson correlation analyses were performed for nutrient content. Results from bacteriological investigations were converted to Log_10_ and graphically presented using GraphPad Prism version 9.2 (GraphPad Software, San Diego, CA, USA). Statistical differences between RMBDs grouped according to storage condition at arrival, and differences in single-protein ABP sources were assessed by two-way ANOVA and Tukey’s multiple comparisons post hoc tests. For all analyses, *p* < 0.05 was considered significant.

## 3. Results

### 3.1. RMBD Detailed Characteristics

A total of 44 RMBDs were evaluated. On arrival, 64% of RMBDs were partly thawed despite being packed in polystyrene boxes and covered with ice. In fact, samples situated in the central part of the box appeared still completely frozen while samples lying at the periphery were defrosted. The mean registered outside temperature during the two shipping days in June 2017 was 18.4 ± 0.1 °C.

The main product characteristics and the composition (ingredient list) obtained from the label are presented in Table 1 and Table 2, respectively.

Most RMBDs (73%) were intended for dogs (*n* = 32, with 3 intended for growth, 15 for adult dogs and 14 proposed for all life stages), 12% were proposed for cats (proposed for all life stages), and 4 out of 44 RMBDs were intended for both dogs and cats (all life stages), as shown in Table 1.

Concerning the type of RMBD, a total of 31/44 RMBDs were marketed as complete pet food (84% for dogs), while among complementary RMBDs, 6 were proposed for dogs and 4 each for cats or both species, respectively (Table 1).

In total, 13/31 complete RMBDs for dogs and cats declared the use of additives on their label. In four complementary products for cats, taurine was added (375 mg/kg fresh matter).

In 13/31 RMBDs clear instructions regarding raw-product handling and storage (“Keep frozen until ready to serve”, “it must be thawed before serving”, “once thawed, it must be stored in fridge and consumed within 24 h”) were given.

A total of 29 RMBDs provided feeding instructions on the label, and for 55% of those products, a suggested daily amount (g/day), based on 2% of the pet’s body weight (BW), was given. Following this instruction, an adult dog of 15 kg BW, with a daily maintenance energy requirement (MER) of 724 kcal ME, would be fed on average only 76% (± 22%) of its MER.

Different sources of meat and animal by-products used in the examined products are shown in Table 2. RMBDs consisted of vacuum-packed ground meat or were sausage-like (plastic tubes sealed with metal clips). Overall, 12 meat sources were declared, with chicken (48%), beef (30%) and salmon (25%) being the most representative ones, followed by duck (16%), rabbit (11%), lamb (9%), whitefish (7%), horse (5%), and goose, turkey, mice, and mackerel (2%). In 27/44 RMBDs, a single meat source was listed on the label, while in the remaining 17 products, two to five different meat sources were included. Most of the samples listed animal by-products (ABPs): offal (91%), bones and cartilage (59%) with 54% of them containing entire animal carcasses (chicken, rabbit, duck, goose, salmon and mice).

Some RMBDs also contained other ingredients, such as vegetables and carbohydrate sources.

### 3.2. Nutrient Content

#### 3.2.1. Analytical Constituents’ Evaluation and Regulation Compliance Assessment

A comparison between declared and measured analytical constituents on an “as is” basis was made (Table 3). In doing so, guidelines about “Tolerances for analytical constituents and additives” (EC No 767/2009, Annex IV, part A) were considered.

Mandatory labelling requirements were not covered in some cases, where data regarding crude fibre (4), crude ash (3), crude protein (2), crude fat (2), and moisture content (1) were lacking (Table 3).

Where possible, compliance with EU regulations was assessed. As shown in Table 3, 95% and 93% of conformity were verified for the declared values for crude fibre and moisture, and 80% of RMBDs were compliant with crude ash content. For crude fat and protein content 33% and 45% of RMBDs, respectively, showed discrepancies. When it came to deviances from labelled information, most of them (fat and ash content and moisture) exceeded the threshold, with the higher percentages of discrepancy for fat and ash content, ranging from 2 to 141% and 3 to 73%, respectively. On the contrary, most relevant discrepancies in crude protein content were verified below the threshold, ranging from 3 to 38% of the labelled values.

#### 3.2.2. Nutrient Content

**Figure 1:**
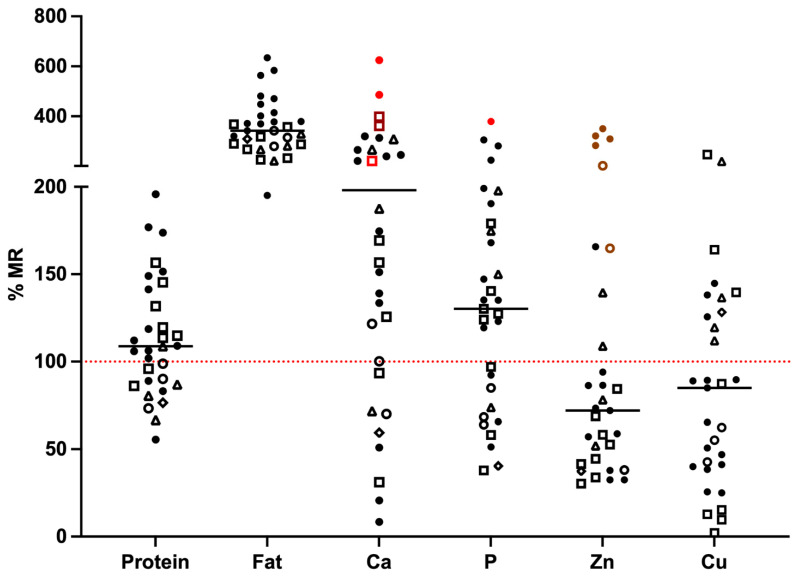
Selected nutrients measured in 31 RMBDs labelled as complete pet food [percent of FEDIAF minimum requirement] according to species and life stage. Full dot—adult dogs (*n* = 15); empty dot—growing dogs (*n* = 3); squared dot—dogs all life stages (*n* = 8); triangle—adult cats (*n* = 4); reverse squared dot—dogs and cats (adult + growth, *n* = 1). Black lines represent the medians. Red dot line represents 100% of FEDIAF minimum requirement according to species and life stage. For calcium and P content, red values exceed the maximum nutritional limit for growth or adult (light red) or both (dark red). For Zn content, brown values exceed the EU legal limit. MR: minimum requirement.

A total of 31 RMBDs, labelled as complete raw pet food, were evaluated for their nutrient content. Protein, fat, calcium, phosphorus, zinc and copper concentration was measured (Table 4) and compared to the minimum requirement (MR) proposed by FEDIAF (2021) for intended species and life stages (Figure 1).

All RMBDs showed at least one nutritional imbalance. The fat content of RMBDs was high (68.9 ± 12.7 g fat/Mcal), in particular when referring to RMBDs proposed for growth (Table 4). The safe upper limit (SUL) for fat according to NRC (2006; 82.5 g fat/Mcal [23]) was exceeded in four RMBDs (three were intended for adult dogs and one for cats). Protein content was below the MR in 26% of RMBDs (mean 84.2 ± 12.0%), and, among the products with protein concentrations below the MR, 37% were proposed for growth (mean 88.8 ± 12.8%). Calcium and phosphorus intake were below MR in 22% (mean 47.3 ± 22.9%) and 32% (mean 70.2 ± 18.4%) of RMBDs, respectively (Figure 1).

Calcium and phosphorus content of RMBDs ranged from 9 to 724% and 43 to 440% of MR, respectively, with an average Ca:P ratio of 1.6 ± 0.6. Among RMBDs proposed for growing dogs, 18% and 45% were inadequate for calcium and phosphorus, respectively (Ca 192 ± 139% MR; P 114 ± 51% MR). A total of five RMBDs exceeded the maximum nutritional limit (N limit) for calcium (two RMBDs proposed for adult dogs and three for all life stages: 8.3 ± 2.0 g Ca/Mcal) and two of those also exceeded (440 and 350%) the N limit of phosphorus (in RMBDs proposed for adult dogs: 5.11 and 4.06 g P/Mcal). Among RMBDs with calcium and phosphorus content above the N limit, all but one product listed bones or entire carcasses in their composition. A Ca:P ratio below 1:1 was found in three RMBDs: two were proposed for adult dogs (0.2:1 and 0.2:1), and one was proposed for all life stages in dogs (0.94:1). A Ca:P ratio above 2:1 was found in seven RMBDs: three were proposed for adult dogs (mean 2.1 ± 0.1), one for growth (2.1) and three for all life stages (mean 2.5 ± 0.6).

Zinc and copper content were insufficient in 68% (mean 62.3 ± 23.1%) and 61% (mean 50.5, SD ± 29.4%) of RMBDs, respectively (Figure 1). However, some products had very high zinc and copper concentrations (up to 358 and 290% MR, respectively), so six RMBDs exceeded the EU legal (L) limit for zinc: four were intended for adult dogs (mean 75.5 ± 9.25 mg Zn/Mcal) and two were intended for growing dogs (44.9 and 58.2 mg Zn/Mcal). In one product intended for all life stages, the L limit for copper was exceeded (8.0 mg Cu/Mcal).

**Figure 2:**
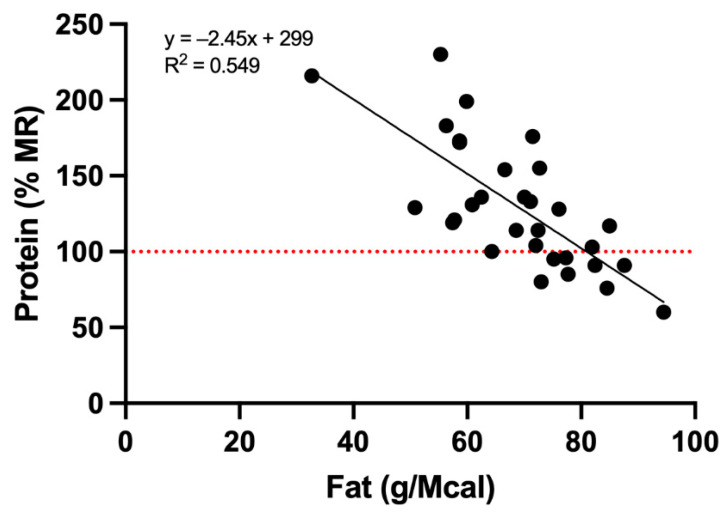
Correlation between fat content (g/Mcal ME) and protein [(percent of FEDIAF minimum requirement) according to species and life stage measured in 31 RMBDs labelled as complete pet food.

Significant negative correlations were found between content of fat (g/Mcal) and other nutrients (expressed as % MR): protein (r = −0.74, *p* < 0.0001; Figure 2), calcium (r = −0.36, *p* = 0.047), phosphorus (r = −0.51, *p* = 0.003), zinc (r = −0.68, *p* < 0.0001), copper (r = −0.37, *p* = 0.038).

### 3.3. Microbial Analysis

**Figure 3:**
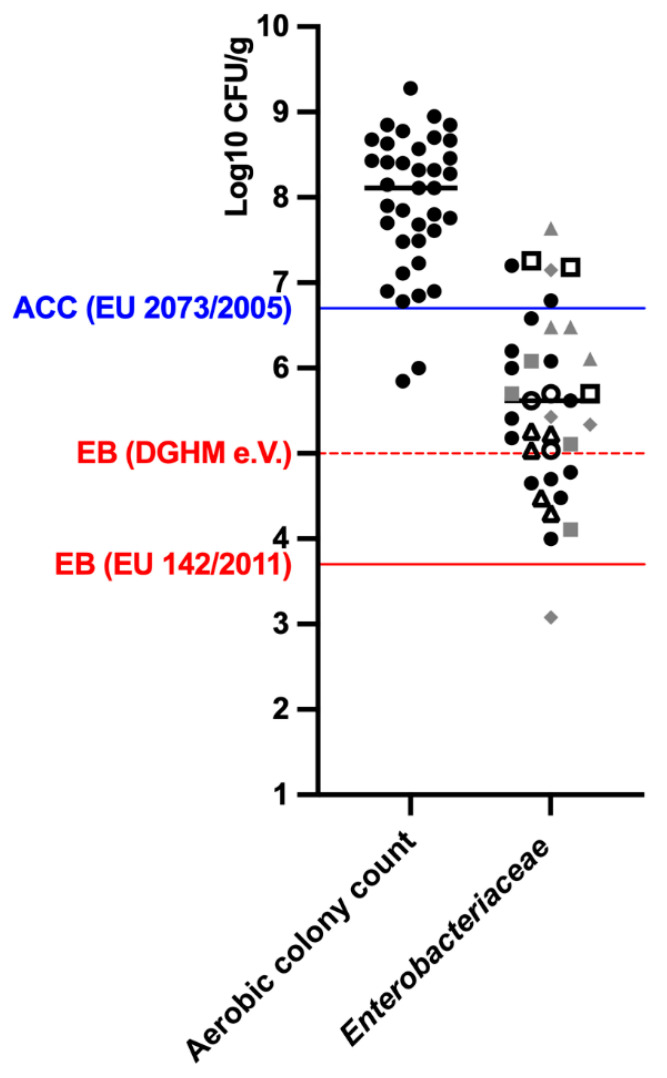
Microbial load (total aerobic bacteria and *Enterobacteriaceae* log_10_ CFU/g) of 37 RMBDs. The blue solid line represents the threshold for aerobic colony count in animal by-products (EC No 2073/2005); the red solid line represents the threshold for Enterobacteriaceae in raw pet food (EC No 142/2011); the red dashed line represents the threshold for *Enterobacteriaceae* in raw beef and poultry meat intended for human consumption (German Society for Hygiene and Microbiology—DGHM e.V.). In relation to *Enterobacteriaceae*, complementary RMBDs are marked in grey and complete RMBDs in black. Full dot—adult dog (*n* = 8); empty dot—puppy (*n* = 1) squared dot—adult and puppy (*n* = 3); triangle—adult cat (*n* = 4); reverse squared dot—dog and cat (adult + growth, *n* = 1). CFU, colony-forming units; ACC, Aerobic Colony Count; EB, *Enterobacteriaceae*.

A total of 37 RMBDs were subjected to microbial quantification analysis for total aerobic colony count (ACC) and *Enterobacteriaceae* (Figure 3). The mean ± SD ACC value was 2.61 × 10^8^ ± 3.63 × 10^8^ CFU/g. Quantitative ACC exceeded the EU unacceptable threshold for hygiene criteria (>5 × 10^6^ = 6.7 log_10_ CFU g; EU 2073/2005 for minced meat and mechanically separated meat intended for human consumption) in all but two RMBDs, ranging from 6 × 10^6^ to 2 × 10^9^ CFU/g. The highest value was found in a beef single-source product. In two RMBDs (one chicken single-source, one mixed animal source) the ACC score was within the marginally acceptable range (5 × 10^5^–5 × 10^6^).

*Enterobacteriaceae* were detected in all RMBDs, with a mean ± SD value of 3.61 × 10^6^ ± 8.39 × 10^6^ CFU/g, exceeding the hygiene criteria for ABPs in raw pet food (5 × 10^3^ = 3.70 log_10_ CFU g/ABPs, EC No 2073/2005) in all but one product. Moreover, 76% of products exceeded the hygiene threshold for *Enterobacteriaceae* (1 × 10^5^ = 5 log_10_ CFU/g) established by the German Society for Hygiene and Microbiology (DGHM e.V.) for raw beef and poultry meat intended for human consumption. Below this threshold, nine RMBDs varied between 1 × 10^4^ and 1 × 10^5^ CFU/g. In total, the *Enterobacteriaceae* load varied from 1.2 × 10^3^ CFU/g (a complementary RMBD containing chicken entire carcasses) to 4.4 × 10^7^ CFU/g (a complementary RMBD consisting of rabbit entire carcasses), with a mean value of 3.61 × 10^6^ CFU/g.

The presence of *Salmonella* spp. was investigated in 10 RMBDs. A positive result was found in 2/10 complete RMBDs from organic farming (chicken and beef single source). In detail, *Salmonella* spp. was detected in one pooled sample by (EIA)-based screening technique (VIDAS), and then confirmed in two individual samples by the cultural reference ISO method.

**Figure 4:**
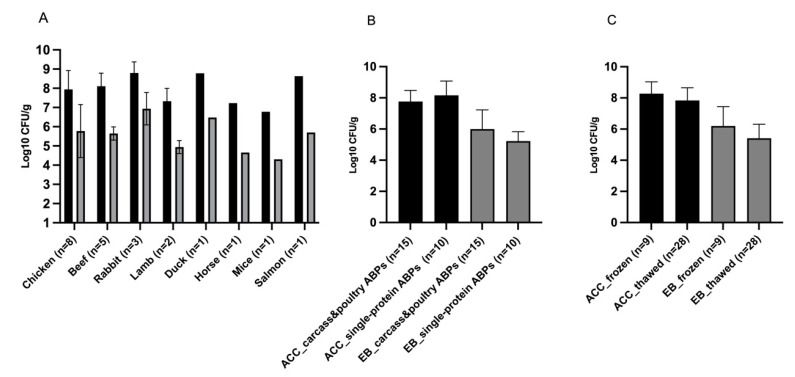
Microbial load (total ACC and *Enterobacteriaceae* Log_10_ CFU/g) of RMBDs grouped according to protein source (**A**), composition (**B**), and storage condition at arrival (frozen or partially thawed) (**C**). Black bars indicate aerobic colony count, grey bars indicate *Enterobacteriaceae* load. ABPs, animal by-products; ACC, aerobic colony count; EB, *Enterobacteriaceae*; CFU, colony-forming units. Error bar is SD.

Figure 4 shows the bacterial load of single-protein ABP products grouped according to protein source (A), composition (B), and storage condition at arrival (frozen or partially thawed) (C). Poultry (chicken *n* = 8 and duck *n* = 1) and beef (*n* = 8) were the most represented single-animal protein sources, and the highest bacterial contamination was found in three RMBDs with rabbit ABPs (Figure 4A): a complete diet for adult dogs (meat and ABPs), a complete diet for dogs of all life stages (entire carcass), and complementary food for cats (entire carcass). In these products, mean ACC and enterobacterial counts were 9.8 × 10^8^ and 2 × 10^7^ CFU/g, respectively. ACC and enterobacterial counts have been grouped according to RMBD composition and storage condition at arrival in Figure 4B,C, respectively. RMBDs containing single-protein ABPs other than poultry or not containing whole carcasses (beef, horse and lamb) were higher in total ACC than RMBDs containing protein ABPs from poultry or entire carcasses from chicken, rabbit, duck, salmon, and mice (8.17 ± 0.91 vs. 7.76 ± 0.71 Log_10_ CFU/g), while the opposite was true for *Enterobacteriaceae* count (6.00 ± 1.23 vs. 5.23 ± 0.60 Log_10_ CFU/g). With regard to storage condition, frozen RMBDs had higher ACC and *Enterobacteriaceae* counts than products partially thawed at arrival (8.27 ± 0.76 vs. 7.83 ± 0.81 and 6.20 ± 1.24 vs. 5.41 ± 0.91 Log_10_ CFU/g, respectively).

Among RMBDs that were still frozen at arrival (Table 5), those containing protein ABPs from poultry or entire carcasses had a significantly higher *Enterobacteriaceae* count than ones containing other single-protein ABPs (7.20 ± 0.14 vs. 5.10 ± 0.21, *p* = 0.009). When compared to RMBDs that were partially thawed at arrival, frozen RMBDs containing protein ABPs from poultry or entire carcasses were higher in *Enterobacteriaceae* count than both counterparts (7.20 ± 0.14 vs. 5.40 ± 0.33, *p* = 0.003), and higher than RMBDs containing other single-protein ABPs (7.20 ± 0.14 vs. 5.28 ± 0.26, *p* = 0.003). No significant differences were found in relation to total ACC (*p* > 0.05, Table 5).

## 4. Discussion

Feeding RMBDs is becoming an increasingly frequent practice among dog and cat owners, and commercial raw pet food is an emerging, convenient, and ready-to-use option to feed raw diets to companion animals. Commercial RMBDs for adult cats and dogs as well as for growing animals are nowadays available as complete or complementary raw pet food. Regulatory compliance for labelling is required for such marketed compound products. Pet food labels provide useful information about products and constitute an essential tool for owners when the decision about how to feed their pet has to be made, even though results from an analysis of pet food label usage have shown that less than 4 out of 10 pet owners regularly check pet food labels [24].

As for traditional pet food, commercial RMBDs manufactured in the EU are subjected to EC No. 769/2009 with regard to labelling regulations. Compliance with EU guidelines in relation to analytical constituents aims to ensure the health of companion animals. In the present study, 9% of RMBDs lacked at least one piece of mandatory information, mainly crude ash and fibre content. In a previous study on Canadian dry dog and cat foods, ash content was not reported in 27% of pet food products [25]. Moreover, among analytical constituents, discrepancies regarding crude protein and fat content were the most represented in RMBDs evaluated in this study (33% and 45%, respectively). More specifically, in 33% of the products, measured fat content was found to be 34% (2–141%) higher than declared. In 19% of the RMBDs, ash content was found to be 33% (3–73%) higher, while in 26%, the measured protein content was 10% (3–38%) lower than declared. Adherence to regulations has been previously verified for traditional pet food with more satisfactory results. In the USA, the difference between labelled and analysed protein content in pet food was acceptable [26], while protein content was below the lower tolerance threshold in 11% of samples of dry pet food sold in Canada [25].

Since it is not mandatory to declare the energy content of pet food in the EU, the organic matter of a product is used to estimate the ME concentration in pet food. Food with a higher energy concentration than expected is especially undesirable in overweight and obese as well as growing animals. Because the daily amount of food is based on its energy content, an inappropriate supply of nutrients can result.

A possible explanation for the determined discrepancies accounting for analytical constituents’ variation might be the lack of standardization of RMBD composition, especially when compared to more processed pet food such as kibbles, as previously demonstrated for wet pet food compared to dry pet food [27]. For the present study, the lower adult energy requirements proposed by FEDIAF for dogs and cats were taken into consideration because they better describe the average energy requirement of neutered and inactive pets [28,29], also considering an increasing prevalence of overweight and obesity [30,31].

In the present study, commercial RMBDs were mostly high in fat (69 (33–95 g/Mcal)) and, consequently, ME. This finding is in agreement with previous studies, in which the fat content of commercial frozen RMBDs was between 64 (dogs) and 92 (cats) g fat/Mcal [32,33]. A nutritional maximum for fat is not provided by the FEDIAF guidelines, but NRC [23] states an SUL of 82.5 g fat/Mcal based on evidence of acute pancreatitis caused in dogs by high-fat diets [34,35]. Even though this relationship has not been confirmed so far, feeding commercial high-fat diets is discouraged in dogs at high risk of developing acute pancreatitis or with an altered lipid status, such as obese dogs [36].

Among RMBDs assessed in this study, 26% of the total and 37% of those proposed for growth, supplied less protein than the MR recommended by FEDIAF [3], which is likely due to their high fat content, as demonstrated by the negative correlation between fat and protein content. Feeding a high-fat, high-energy, low-protein diet either leads to deficient protein (and amino acids) intake or energy excess. Mineral imbalances were identified even though RMBDs were labelled as complete pet food. RMBDs containing only meat and meat by-products caused insufficient Ca and P intake while some products containing bones exceeded the nutritional maximum for those minerals.

Veterinarians and owners should therefore be aware that feeding RMBDs without mineral sources in their composition list may lead to mineral deficiencies and should not be considered appropriate for growing animals and long-term feeding of adults [37]. Clinical diffuse osteopenia has been reported in growing dogs and cats fed raw diets [38,39,40,41], improving after balancing the diet.

A deficient as well as excessive supply of Ca and P can cause developmental orthopaedic diseases [42,43,44], especially in large breed dogs [45,46]. An evaluation of the supply has to take the availability of the minerals into account, which depends on factors such as the amount and sources, fibre content and digestibility of dry matter [47,48].

In the present study, three RMBDs intended for adult and growing dogs exceeded the nutritional maximum of Ca for canine growth (ranging from 260 to 467% of MR) and two out of three also exceeded the calcium N maximum for the adult phase (ranging from 358 to 644% of MR). In a previous study, Beagle puppies fed a diet providing 300% of Ca MR, over a period of 21 weeks (from 6 to 27 weeks of age), showed signs of impaired skeletal development, related to Ca excess, without developing pain or lameness [46]. Therefore, possible impairment of skeletal development in growing dogs fed commercial unbalanced RMBDs rich in bones must be considered, especially for large and medium breed dogs.

In this study, 68 and 61% of commercial RMBDs that were evaluated failed to cover MR for Zn and Cu, respectively. The high Ca and P content of some RMBDs can further reduce trace element absorption. One RMBD was very high in Cu (300% MR), probably originating from liver labelled as lamb offal. Dietary Cu excess has recently been of concern because of the perceived increase in Cu-associated hepatopathy in dogs [49].

In the present study, six RMBDs (four for adult dogs, two for growth) exceeded the legal maximum for Zn. All these products declared an addition of Zn sulfate monohydrate ranging from 39 to 50 mg Zn/kg, while the analysed concentration was two to four-fold higher than this. It is important to note that the legal maximum of a nutrient relates to the total amount in the product.

The RMBDs consist of APBs from Category 3 material (EC No 1069/2009, Article 10a, b(i)(ii)), which are not intended for human consumption [50]. EC No 142/2011 regulates microbial contamination in pet food: for raw pet food, in particular, it does not allow the presence of Salmonella in five samples and limits the number of *Enterobacteriaceae* spp. in raw pet food to <5000 CFU/g in 2/5 samples of a tested batch (Annexes X-XIII, [22]). No EU regulations on maximum limits of ACC have been proposed for pet food so far; therefore, in this study, ACC levels considered as a measure of process hygiene were evaluated according to regulation on microbiological food safety and process hygiene criteria for foodstuffs (EC No 2073/2005).

The presence of *Enterobacteriaceae* is linked to contamination with faeces or intestinal content, indicating poor hygiene procedures in the slaughterhouse, inappropriate storage, or both [51]. Moreover, contamination with *Enterobacteriaceae* may play a role in meat spoilage due to their ability to metabolize amino acids into malodorous volatile compounds such as diamines and sulphuric compounds [51,52]. In this study, *Enterobacteriaceae* were detected in all RMBDs, with a prevalence similar to previous findings, ranging from 78% to 100% of RMBDs [10,14,15]. However, samples from the present study had the highest microbial load from *Enterobacteriaceae* reported in RMBDs so far, with a mean score about 2 log_10_ CFU/g higher than findings in other studies [10,15]. All but one RMBD in this study exceeded the hygiene limit for ABPs, according to European legislation, and 76% of them failed to meet hygiene criteria according to the German Society for Hygiene and Microbiology (DGHM e.V.). The latter criteria are for poultry and beef meat for human consumption, thus, contrary to feeding raw pet food, a cooking process before consumption can be assumed. Such high levels of contamination may pose a health risk for dogs and cats especially after storage of the material, because enterobacteria levels of 10^7^ CFU/g can lead to spoilage of poultry products, even during refrigeration [52]. In this study, the highest levels of *Enterobacteriaceae* were found in RMDBs containing single-protein ABPs or carcasses from rabbits (*n* = 3) and ducks (*n* = 3). Unfortunately, data in the literature regarding microbial contamination of rabbit meat and carcasses, compared to red meat or poultry, are scarce. Compared to our findings, studies from Italy and Spain described notably lower mean enterobacteria contamination of rabbit carcasses intended for human consumption (<10^3^ CFU/cm^2^ and 0.5 log CFU/g, respectively) [53,54].

When evaluated in relation to the storage condition at arrival and the different animal sources, significant differences in *Enterobacteriaceae* contamination were recorded. In fact, microbial contamination was significantly higher in frozen RMBDs containing protein ABPs from poultry or entire carcasses than in RMBDs from different animal protein sources, regardless of the storage condition at arrival. In this regard, more intense soiling with faeces and intestinal content can be expected in specific animal sources, such as poultry and rabbit [55].

Interestingly, frozen RMBDs containing protein ABPs from poultry or entire carcasses had higher enterobacterial contamination than the partially thawed counterpart. It is known that certain bacteria belonging to *Enterobacteriaceae* may exert a mechanism of survival resistance when exposed to harsh environmental conditions or sub-lethal stress, such as freeze–thaw processes [56,57]. An underestimation of viable *Enterobacteriaceae* in partially thawed RMBDs can therefore be hypothesized.

Belonging to enterobacteria, *Salmonella* spp. is a well-known zoonotic bacterial pathogen, often occurring in products of animal origin, mainly in poultry and beef meat. In this study, *Salmonella* spp. was detected in 20% of the selected samples, in accordance with results from other studies [11,12]. However, data regarding the prevalence of *Salmonella* spp. in RMBDs are very variable, ranging between 0% and 71% [10,13,15,58,59]. Controlling *Salmonella* on raw meat and poultry is considerably complex, and due to the zero-tolerance policy established by the EU regulation, it has been notified as the main bacterial food, feed materials and pet food hazard in the EU market since 1980 [60].

In the present study, *Salmonella* spp. was isolated only in one chicken and one beef RMBD labelled as “organic” raw pet food. Organic pet food may be perceived as an alternative to conventional pet food for those consumers who want to avoid certain potentially harmful contaminants [61], even though this held not true for these products.

In the present study, mean total ACC was 2.61 × 10^8^ (±3.63 × 10^8^) CFU/g, with an unacceptable hygiene quality in 96% of RMBDs when limits for products for human consumption are considered. In recently published European studies, total ACC measured in RMBDs showed a wide range of bacterial contamination, from 7.9 × 10^2^ to 7.4 × 10^8^ CFU/g [11,13,15]. The high total ACC recorded in the present study is similar to the findings of a recent study from Thailand, where a median ACC score of 1.81 × 10^8^ CFU/g was measured in 17 RMBDs [62]. High total ACC may cause rapid alteration of meat during cold storage due to microbial growth, protein degradation, and lipid oxidation. Counts between 10^7^ and 10^8^ CFU/g are generally associated with changes in organoleptic properties such as appearance, smell, and taste [63]. However, high levels of ACC do not necessarily pose risks for animal or human health, as food spoilage is not correlated with ACC growth [64]. High ACC levels may be due to heavy contamination of raw ABPs. In fact, most RMBDs evaluated in the present work included offal such as stomach, intestine, and beef tripe, not intended for human consumption and, for this reason, probably not subjected to strict hygiene controls and procedures. This could explain the high ACC levels that were detected in RMBDs containing ABPs specifically from poultry, beef, and rabbit, as previously reported for RMBDs sold in other European countries [15,58]. Poor storage conditions may affect the microbial load; however, in this study, microbial load was not higher in the 64% of RMBDs that were, at least externally, partially defrost on arrival. In this regard, Morelli et al. (2020) described an increase of 1.5 log_10_ CFU/g of ACC microbial load in RMBDs stored at 2 °C for 3 days, advising that RMBDs should be consumed within 24 h after defrosting [58].

Results from this study highlight the potential health risk to cats and dogs originating from feeding RMBDs, even though a broad microbial analysis was not performed and not all RMBDs were screened for *Salmonella* spp.

The potential zoonotic risk for disease transmission from pets being fed with RMBDs to humans is a concern as well, despite the low frequency of suspected or confirmed self-reported pathogen transmission—around 0.2% from recent surveys [65,66]. Pathogens can be spread between pets and humans in the same household by being contaminated with faeces [67] or inappropriate handling and storage procedures of RMBDs [8]. It is necessary, from a One Health perspective, to keep the public interest up on the potential health risk of feeding RMBDs to dogs and cats.

## 5. Conclusions

Feeding commercial RMBDs transported and stored frozen is the desired solution for owners who want to avoid feeding their pets otherwise processed foods. However, this study highlights issues related to commercial RMBDs: (i) evaluated commercial RMBDs showed considerable discrepancies between analysed and declared nutrient content, causing deficient and/or excessive concentrations of protein, fat and certain minerals when compared to recommended daily allowances, and (ii) commercial RMBDs containing entire animal carcasses and by-products had poor microbiological quality.

## Figures and Tables

**Table 1:** Selected labelled information on purchased RMBDs (*n* = 44).

**Animal species ^§^:**	
Dog	32
Cat	8
Both	4
**Life stages (D: dog; C: cat; B: both):**	
Maintenance	15 (D); 4 (C)
Growth	3 (D)
All phases	14 (D); 4 (C); 4 (B)
**Type of RMBD (D: dog; C: cat; B: both) ^§^:**	
Complete	26 (D); 4 (C); 1 (B)
Of these: with additives declared	11 (D); 3 (C)
Complementary	6 (D); 4 (C); 3 (B)
**Hygiene and/or handling advice:**	
Yes	13
No	31
**Feeding instructions (daily ration) ^§^:**	
Yes	29
No	15

^§^ legally required (EC No 767/2009).

**Table 2:** Meat and animal by-products sources in RMBDs (*n* = 44).

Main Meat Source	Meat	Offal	Bones & Cartilage	Entire Carcass
Chicken (*n* = 21)				
Single-meat source (7/21)	5	5	4	2
Multi-meat sources (14/21)	12	13	13	1
Beef (*n* = 13)				
Single-meat source (8/13)	8	7	1	/
Multi-meat sources (5/13)	3	3	/	/
Salmon (*n* = 11)				
Single-meat source (1/11)	/	/	/	1
Multi-meat sources (10/11)	/	/	/	10
Duck (*n* = 7)				
Single-meat source (3/7)	1	1	1	2
Multi-meat sources (4/7)	3	3	2	/
Rabbit (*n* = 5)				
Single-meat source (3/5)	1	1	1	2
Multi-meat sources (2/5)	1	2	1	/
Lamb (*n* = 4)				
Single-meat source (2/4)	2	2	1	/
Multi-meat sources (2/4)	2	1	1	/
Whitefish (*n* = 3)				
Single-meat source (0/3)	/	/	/	/
Multi-meat sources (3/3)	/	/	/	3
Horse (*n* = 2)				
Single-meat source (1/2)	1	1	/	/
Multi-meat sources (1/2)	1	1	/	/
Goose (*n* = 1)				
Single-meat source (1/1)	/	/	/	1
Multi-meat sources (0/1)	/	/	/	/
Mackerel (*n* = 1)				
Single-meat source (0/1)	/	/	/	/
Multi-meat sources (1/1)	/	/	/	1
Mice (*n* = 1)				
Single-meat source (1/1)	/	/	/	1
Multi-meat sources (0/1)	/	/	/	/
Turkey (*n* = 1)				
Single-meat source (0/1)	/	/	/	/
Multi-meat sources (1/1)	1	1	1	/

**Table 3:** Comparison of declared vs. measured analytical constituents in RMBDs in % aberrancy beyond legally tolerated concentrations (EC No 767/2009; Annex IV, part A).

Constituent	Number of Analysed RMBDs	RMBD (*n*) Compliance with EU Regulation ^§^	Discrepancies Detected	
			Below	Above
Crude fat	42	23	5	14
aberrancy [%] median (range)			28.7 (2.0–70.1)	33.7 (2.1–141.3)
Crude protein	42	28	11	3
aberrancy [%] median (range)			10.2 (3.3–38.2)	9.9 (2.8–17.0)
Crude ash	41	33	0	8
aberrancy [%] median (range)				33.4 (3.2–72.9)
Crude fibre	40	38	2	0
aberrancy [%] median (range)			70.9 ± 19.0	
Moisture	43	40	0	3
aberrancy [%] median (range)				13.3 (3.3–15.7)

^§^ Crude fat: <16%, 4% absolute (abs.) above and 2% abs. below; 16–24%, 25% abs. above and 12.5% abs. below; crude protein: <16%, 2% abs. above and below; 16–24%, 12.5% relative (rel.) above and below; >24%, 3% abs. above and below; crude ash: <8%, 2% abs. below and 1% abs. above; crude fibre: <10%, 1.75% abs. below and above; moisture: >12.5%, 8% rel. above and none below.

**Table 4:** Macronutrient, selected minerals and energy content [mean ± SD; range (min-max)] of 31 RMBDs labelled as complete pet food.

	Dogs and Cats	Dogs			Cats	Dogs and Cats
Nutrient/Mcal	Overall (*n* = 31)	Adult (*n* = 15)	Growth (*n* = 3)	All Life Stages (*n* = 8)	Adult and Growth (*n* = 4)	All life Stages (*n* = 1)
Crude protein, g	77.2 ± 22.4	74.0 ± 25.1	60.3 ± 10.6	86.0 ± 19.9	85.7 ± 20.8	71.0
	31.3–120	31.3–120	49.9–71.2	56.8–114.0	63.2–113.0	
Crude fat, g	68.9 ± 12.7	65.4 ± 15.7	72.9 ± 4.35	70.4 ± 9.7	73.6 ± 9.1	77.7
	32.7–94.5	32.7–94.5	68.6–77.3	56.3–82.4	62.5–84.6	
Calcium, g	3.9 ± 2.7	3.7 ± 2.8	2.7 ± 0.8	4.5 ± 3.0	4.8 ± 2.4	1.3
	0.1–10.5	0.1–10.5	1.9–3.5	0.7–9.3	1.6–7.0	
Phosphorus, g	2.3 ± 1.1	2.3 ± 1.3	1.8 ± 0.2	2.2 ± 0.9	2.9 ± 1.1	0.9
	0.6–5.1	0.6–5.1	1.7–2.0	0.7–3.7	1.3–3.8	
Ca:P ratio	1.6 ± 0.6	1.5 ± 0.6	1.5 ± 0.5	1.9 ± 0.7	1.6 ± 0.3	1.4
	0.2–2.3	0.2–2.3	1.1–2.1	0.9–3.2	1.1–1.8	
Copper, mg	2.2 ± 1.7	1.7 ± 0.9	1.6 ± 0.3	2.7 ± 2.9	2.6 ± 1.2	3.9
	0.1–8.0	0.6–3.4	1.3–2.0	0.1–8.0	1.3–4.2	
Zinc, mg	27.2 ± 22.5	32.6 ± 28.1	37.7 ± 24.8	14.9 ± 5.7	27.1 ± 10.7	10.4
	7.3–88.9	7.3–88.9	9.5–50.5	8.0–24.7	15.7–39.8	
ME, kcal/kg	1825 ± 527	1855 ± 676	1936 ± 323	1773 ± 451	1732 ± 266	1833
	976–3701	976–3701	1578–2206	1179–2551	1574–2128	

ME: metabolizable energy.

**Table 5:** Effects of the storage condition at arrival and specific animal source of RMBDs on aerobic colony and *Enterobacteriaceae* counts.

	Frozen (*n* = 8)		Partially Thawed (*n* = 17)		ANOVA *p*-Value		
	Carcass and Poultry (*n* = 5)	Single-Protein ABPs (*n* = 3)	Carcass and Poultry (*n* = 10)	Single-Protein ABPs (*n* = 7)	Storage Condition	Ingredients	Interaction
Aerobic colony count, Log_10_ CFU/g	8.69 ± 0.27	7.64 ± 0.42	7.91 ± 0.30	7.81 ± 0.29	0.404	0.128	0.200
*Enterobacteriaceae*, Log_10_ CFU/g	7.20 ± 0.14 ^a^	5.10 ± 0.21 ^b^	5.40 ± 0.33 ^b^	5.28 ± 0.26 ^b^	0.033	0.005	0.010

Data given as mean ± SEM. Means with different superscripts are significantly different in multiple comparisons.

## Data Availability

Not applicable.
